# Advances in Plant Antiviral RNAi: From Host DCLs/RDRs to Diversified Viral Counteracting Strategies

**DOI:** 10.3390/v18020184

**Published:** 2026-01-29

**Authors:** Xue Li, Fuan Pan, Xueping Zhou, Aiming Wang, Richard Kormelink, Fangfang Li

**Affiliations:** 1 State Key Laboratory for Biology of Plant Diseases and Insect Pests, Institute of Plant Protection, Chinese Academy of Agricultural Sciences, Beijing 100193, China; 2 London Research and Development Centre, Agriculture and Agri-Food Canada, London, ON N5V 4T3, Canada; 3 Laboratory of Virology, Wageningen University and Research, 6700 AA Wageningen, The Netherlands

**Keywords:** plant RNA interference, DICER-like endonucleases, RNA-dependent RNA polymerase, viral RNA silencing suppressors, XRN-resistant non-coding RNA, antiviral immunity

## Abstract

Plant RNA interference (RNAi) is a fundamental antiviral defense that relies on coordinated activities of DICER-like endonucleases (DCLs), Argonaute proteins (AGOs) and RNA-dependent RNA polymerases (RDRs). Over the past decades, studies using model and crop species have uncovered complex and often redundant roles for DCLs and RDRs in generating and amplifying virus-derived small interfering RNAs (vsiRNAs), in addition to connections with transcriptional gene silencing (TGS) and epigenetic defenses against DNA viruses. Concurrently, plant viruses have evolved diverse counterstrategies—proteinaceous RNA silencing suppressors (RSSs), exoribonuclease (XRN)-resistant noncoding RNAs, and indirect manipulation of host pathways—to evade RNAi. Driven by the co-evolutionary arms race, plants have developed sophisticated counter-countermeasures that modulate or overcome viral anti-RNAi activity. Accumulated evidence suggests that plants encode host factor genes that are activated to degrade or sequester viral components such as RSSs against viral infection. On the other hand, plants have also evolved endogenous host modulators of antiviral RNAi that can either reinforce the antiviral response or be co-opted by viruses to antagonize it, representing a furious dynamic molecular battling mechanism. Here, we review recent advances in the molecular functions of DCLs and RDRs across species, summarize newly discovered viral counter-defenses (including RNA-based suppressors), and discuss host counter-countermeasures. We research key areas—such as the roles of RDRγ-class proteins, RTL1 (RNase three-like 1)-mediated competition with DCLs, and the mechanistic impact of viral noncoding RNAs—and outline translational opportunities for improving virus resistance in crops through breeding, biotechnological approaches, and RNA-based applications.

## 1. Introduction

Plants are persistently challenged by diverse DNA and RNA viruses that threaten agricultural productivity and ecosystem health. To counter these threats, plants deploy RNA interference (RNAi) as a frontline, sequence-specific antiviral defense [1,2,3,4]. RNAi integrates small RNA biogenesis, effector loading and target cleavage/transcriptional silencing, thereby reducing viral RNAs and, in the case of some DNA viruses, directing epigenetic modifications of viral genomes. The core molecular machinery—DICER-like endonucleases (DCLs), Argonaute proteins (AGOs) and RNA-dependent RNA polymerases (RDRs)—has been studied extensively in model species such as *Arabidopsis thaliana* and in key crops, revealing both conserved principles and species-specific specializations.

Despite the apparent simplicity of the “DCL → vsiRNA → AGO” paradigm, antiviral RNAi constitutes a complex, multilayered network [1,2,3,4,5]. Different DCL paralogs generate discrete vsiRNA size classes (e.g., 21–24 nt) with distinct antiviral efficacies; RDRs amplify primary signals into secondary siRNAs and enable systemic spread; and cross-talk between post-transcriptional gene silencing (PTGS) and transcriptional gene silencing (TGS) links small RNA pathways to DNA methylation—an important defense against DNA viruses such as those of the *Geminiviridae*. Moreover, multiple endogenous regulators (e.g., Gene Silencing 3 (SGS3), RNase three-like 1 (RTL1) and certain calmodulin-like proteins) and cellular processes such as autophagy, proteasomal degradation, and the unfolded protein response (UPR), modulate the activity and stability of RNAi components, thereby adding further regulatory layers. In addition, plant defense strategies are not only rooted in resistance but also in tolerance mechanisms, which provide additional dimensions to the overall defense network [6].

Co-evolving with host defenses, viruses have mounted sophisticated counterstrategies to establish infection, ranging from expression of proteinaceous RNA silencing suppressors (RSSs) that sequester dsRNA/siRNA or degrade RNAi components to the production of exoribonuclease (XRN)-resistant noncoding RNAs and manipulation of host protein degradation or methylation pathways. As discussed recently [7], the plant–virus arms race extends beyond RNAi, incorporating additional RNA layers of host–virus interactions. Importantly, hosts in turn can evolve or deploy factors that neutralize viral RSSs. Given this dynamic arms race, a current challenge is to integrate mechanistic insights into DCL and RDR biology with an understanding of viral countermeasures to design effective, durable resistance. Therefore, this review focuses on recent discoveries regarding the molecular roles of DCLs and RDRs in antiviral defense, the expanding repertoire of viral counterstrategies (including RNA-based suppressors), and host factors that modulate these interactions, and concludes with perspectives on unresolved questions and translational potential.

## 2. The RNAi Core Machinery: From DCL Cleavage to RDR Amplification in Antiviral Pathways

The mechanisms underlying RNAi have been studied extensively by many research laboratories in the past two decades, especially the functions of core RNAi effectors. Using *Arabidopsis thaliana* as a model plant, many antiviral RNAi effectors have been identified [8,9,10,11,12,13,14,15] (Table 1 and Table 2), and proteins in three families, namely DCLs, AGOs, and RDRs, are now considered as the core effectors of RNAi that act in coordination to regulate viral DNA or RNA accumulation in infected plants.

### 2.1. Core Antiviral RNAi Effectors: DCLs

**Figure 1:**
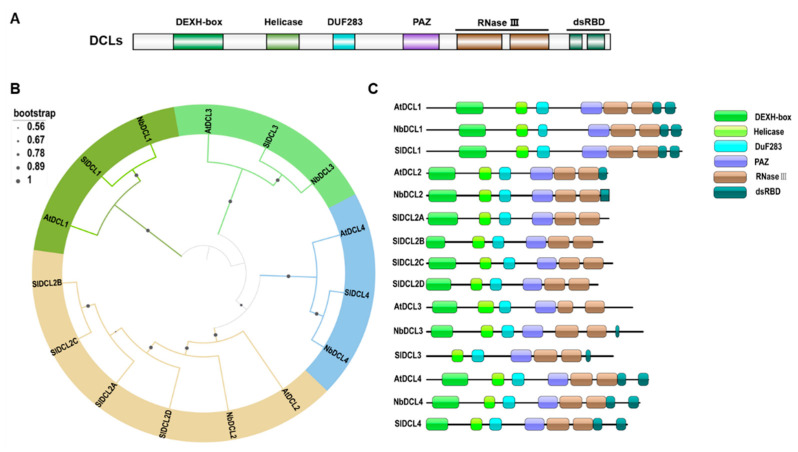
Phylogenetic relationships among *Arabidopsis thaliana*, *Nicotiana benthamiana*, and *Solanum lycopersium* DCLs and their conserved domains. (**A**) Diagram of the domain organization of the plant DCLs. (**B**) A neighbor-joining tree was constructed after multiple alignments using *A. thaliana*, *N. benthamiana*, and *S. lycopersium* DCL protein sequences. The bootstrap values calculated using 1000 replicates are indicated. Four different colors mark the four *DCL* gene families. (**C**) Schematic diagrams showing the domain organizations of individual DCL proteins. The SMART online tool (https://smart.embl.de/, accessed on 15 December 2025) was used to predict the conserved domains in these DCLs. DEXH-box, DEAD box helicase domain; Helicase, C-terminal helicase domain; DUF283, a domain of unknown function 283; PAZ, a Piwi/Argonaute/ZWILLE domain; RNase III, Ribonuclease III domain; dsRBD, dsRNA binding domain.

To look into discoveries regarding how RNAi counters viral infection, we first review the DCL family—their domain architecture, substrate specificity, and experimentally defined antiviral functions across model and crop species. The essential components of small RNA biogenesis are conserved among all plant species [16,17,18]. For example, the DCLs are the endonucleases from the RNase III family that recognize and process dsRNAs. Plant DCLs are known to contain several domains, including a DexD/H (Asp-Glu-X-Asp/His) box, a helicase domain, a DUF283 domain (domain of unknown function), a Piwi/Argonaute/ZWILLE (PAZ) domain, an RNaseIII domain, and one or more dsRNA binding domains (dsRBDs) (Figure 1A). Depending on the specific functional domains such as PAZ, DExD/H, and dsRBDs, DCLs recognize and target specific dsRNA substrates for further processing. The small RNA processing mode that involves the PAZ domain binding to the 2-overhang nucleotides (nt) at the 3′ end of the dsRNA precursors is the primary mechanism used by DCLs during the biogenesis of both siRNAs and microRNAs (miRNAs). The substrate dsRNAs are then extended along the surface of DCLs till the cleft is formed by two, dimerized RNaseIII domains, and then cleaved by the two corresponding active sites to produce staggered RNA duplex ruptures. The enzymatic complex structure seems to be a ruler determining the size of the small RNA [19]. DCLs in *A. thaliana*, *Nicotiana benthamiana*, and *Solanum lycopersium* share high amino acid sequence identities. They have highly conserved domain organizations (Figure 1B,C). Four DCLs are known to be functional in *A. thaliana* (i.e., AtDCL1, AtDCL2, AtDCL3, and AtDCL4) [11] and four DCLs in *N. benthamiana* (i.e., NbDCL1, NbDCL2, NbDCL3, and NbDCL4) are known to be functional, according to their conserved domain organizations [20,21,22,23,24]. In 2012, seven DCLs (i.e., SlDCL1, SlDCL2A, SlDCL2B, SlDCL2C, SlDCL2D, SlDCL3, and SlDCL4) were predicted in *S*. *lycopersium*, and five of them (i.e., SlDCL1, SlDCL2A, SlDCL2B, SlDCL3, and SlDCL4) have been functionally characterized [16].

**Figure 2:**
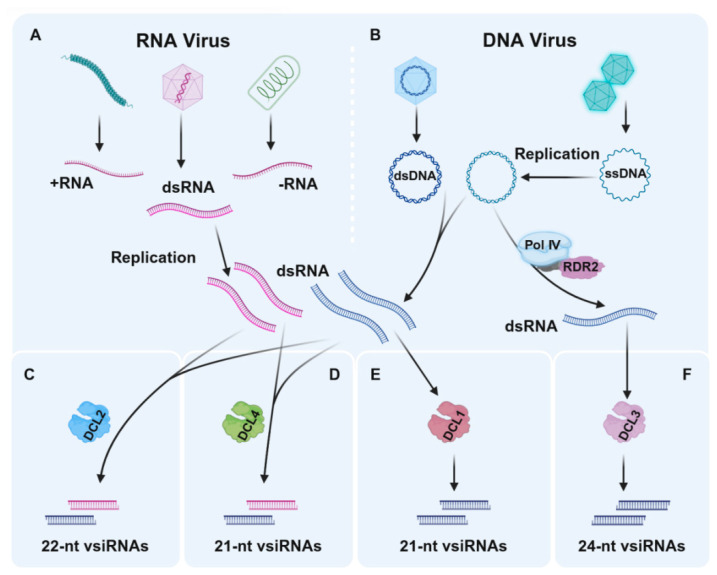
DCLs in plant antiviral RNA interference. (**A**) The generation of dsRNA by RNA viruses. (**B**) The process by which DNA viruses produce dsRNA. (**C**) DCL4 generates the dominant 21-nt vsiRNAs, which are most effective in antiviral defense. (**D**) DCL2-generated 22-nt vsiRNAs, which are less efficient in RNAi induction. (**E**) DCL1 contributes to the production of 21-nt vsiRNAs from DNA viruses [25,31]. (**F**) DCL3 produces 24-nt siRNAs in DNA virus-infected plants, contributing to antiviral RNAi. +RNA, Positive-sense single-stranded RNA; −RNA, Negative-sense single-stranded RNA; dsRNA, double-stranded RNA; dsDNA, double-stranded DNA; ssDNA, single-stranded DNA; Pol Ⅳ, RNA polymerase IV; RDR2, RNA-dependent RNA polymerase 2; DCL, DICER-like endonuclease; vsiRNAs, virus-derived small interfering RNAs. The purple RNA originates from RNA viruses, while the blue RNA originates from DNA viruses. Image created using BioRender.com, with permission (https://app.biorender.com/illustrations/6942753daf6371d00902435b, accessed on 28 December 2025).

Antiviral RNAi is triggered by the recognition of highly structured or double-stranded viral RNAs (dsvRNAs), and these dsRNA are processed into 21–24 nt vsiRNAs by DCLs. A wealth of evidence demonstrates that the DCLs can function redundantly or cooperatively to produce vsiRNAs to confer host resistance to virus infections [11,12,19,25,26,27]. For example, *A. thaliana dcl* single or double knockout mutant lines are hyper-susceptible to viral infections, accumulate more viral RNAs and proteins, and display more severe disease symptoms than the virus-infected wild-type *A. thaliana* plants [11,14]. The DCL4-generated 21-nt vsiRNA is the dominant species among the vsiRNAs that can direct potent antiviral defense. DCL4 can also repress the accumulation of DCL2-generated 22-nt vsiRNA, which is less effective in the induction of RNAi than the 21-nt vsiRNA [11,14] (Figure 2A,B). DCL3 can generate high levels of 24-nt siRNA in DNA virus-infected *A. thaliana* plants [25,28,29] (Figure 2D), which is crucial for the methylation-mediated antiviral defense against geminivirus infections [29]. DCLs have also been reported to play important functions in other plant–virus interactions. For example, Kwon and his colleagues generated multiple *dcl2* and *dcl4* double knock-down tomato plants and found that DCL2, DCL4, or both can induce host resistance to potato virus X (PVX) and potato virus Y (PVY) infections [30]. In contrast, DCL1 can repress antiviral RNAi through the negative regulation of *DCL4* and *DCL3* [15], even though its primary function is to produce miRNAs [31], and the production of 21-nt vsiRNA from different DNA viruses (Figure 2C), e.g., cabbage leaf curl virus (CaLCuV) and cauliflower mosaic virus (CaMV) [25]. A recent study reported on the discovery of tomato SlDCL2b affecting the biogenesis of 22-nt miRNAs, and interfering with the production of 22-nt secondary siRNAs from antiviral host defense genes [32]. Notably, SlDCL1 can produce canonical miRNAs and some 21-nt siRNAs, while SlDCL3 can produce heterochromatic 24-nt siRNAs and long miRNAs [33,34]. SlDCL2A/B are known to produce 22-nt endogenous sRNAs, including miRNAs, and to exhibit host defense against RNA virus infections [32,35]. SlDCL4 can regulate tomato leaf development by producing 21-nt trans-acting siRNAs (tasiRNAs) to regulate host auxin response by targeting auxin-responsive factors [36].

Another group of dsRNA nucleases are RTL enzymes, which lack the DCL-specific domains, and their functions in virus infection in *A. thaliana* plants have been documented [37]. RTL1 evolved as a cellular defense factor that helps plants to fight against viruses, likely by degrading viral dsRNA replication intermediates. In vivo and in vitro assays have confirmed that RTL1 can block siRNA production through cleavage of dsRNAs prior to DCL2-, DCL3-, and DCL4-mediated processing. Therefore, RTL1 is considered a competitor of antiviral PTGS to prevent secondary siRNA amplification, from which virus replication may benefit. However, and intriguingly, virulent viruses like cucumber mosaic virus (CMV), turnip crinkle virus (TCV), turnip mosaic virus (TuMV), and turnip vein clearing virus (TVCV) have evolved strategies to inhibit RTL1 activity and antiviral PTGS at the same time. On the other hand, some viruses, like turnip yellow mosaic virus (TYMV), do not appear to impede RTL1 activity and, consequently, RTL1 competes with antiviral RNAi to degrade viral-derived dsRNA. As such, the strong RNAi defense pathway is abolished, and TYMV can still replicate to some extent, indicating that antiviral RNAi is more effective at destroying viral dsRNAs in the absence of RTL1-mediated cleavage of viral dsRNAs.

Studies have also reported on potential DCL-independent production of siRNAs [38], in which their biogenesis results from trimming of larger dsRNA molecules by 3′-to-5′ exonucleases. This population shows up as a “ladder” in a size range of 20–60 nt, in which all siRNAs shared a 5′-end but differ at their 3′-ends. However, the existence of this pathway is being debated. Having outlined how DCLs generate vsiRNAs, we next consider AGOs—the effectors that load small RNAs and execute target repression at post-transcriptional or transcriptional levels.

**Table 1:** Plant DCLs/RTL1 and their functions in virus infections. BCTV, beet curly top virus; CaLCuV, cabbage leaf curl virus; CaMV, cauliflower mosaic virus; CMV, cucumber mosaic virus; ORMV, oilseed rape mosaic virus; PVX, potato virus X; PVY, potato virus Y; ToMV, tomato mosaic virus; TRV, tobacco rattle virus; TCV, turnip crinkle virus; TuMV, turnip mosaic virus; TVCV, turnip vein clearing virus; TYMV, turnip yellow mosaic virus. *At*, *Arabidopsis thaliana*; *Nb*, *Nicotiana benthamiana*; *Sl*, *Solanum lycopersium*.

DCL/ RTL	Virus	Host	Main Functions	Reference
DCL1	TCV	*At*	DCL1 down-regulates the expression of DCL4 and DCL3, to negatively affect antiviral RNAi	[15]
	CaLCuV		DCL1 generates 21-nt vsiRNAs of DNA viruses (DCL1 mainly functions in the biogenesis of miRNAs)	[25,39]
	CaMV			
DCL2	TRV	*At*	DCL2 is required for the biosynthesis of 22-nt vsiRNAs, and mediates antiviral RNAi (DCL2 usually functions when DCL4 is suppressed or in the absence of virus RNA silencing suppression)	[11,12]
	TuMV			[14]
	CMV			[8]
	CaLCuV			[25,28,39]
	CaMV			
	ORMV			
	PVX		DCL2 contributes to the suppression of viral systemic infection	[21]
	PVX	*Sl*	DCL2 contributes to tolerance to virus infection	[30]
	PVY			
	ToMV		DCL2 affects the biosynthesis of 22-nt miRNAs, thus regulating host defense genes to affect virus immunity	[32]
DCL3	CaLCuV	*At*	DCL3 is the enzyme most associated with methylation-mediated defense through generating 24-nt vsiRNAs	[29]
	BCTV			
	CaLCuV	*At*	DCL3 affects the biosynthesis of 24-nt vsiRNAs (DCL3 mainly functions in the plant–DNA virus interaction, and shows a weak activity in the dicing 24-nt vsiRNAs of plant RNA viruses)	[28]
	CaLCuV			[25,39]
	CaMV			
	ORMV			
	TRV			[12]
DCL4	CMV	*At*	DCL4 is required for the biosynthesis of 21-nt vsiRNAs, and mediates antiviral RNAi defense (DCL4 is the primary DCL component of antiviral defense against RNA viruses)	[26]
	TRV			[11,12]
	CaLCuV			[25,39]
	CaMV			
	ORMV			
	TuMV			[14]
	PVX	*At* *Nb*	DCL4 contributes to the suppression of viral replication and systemic infection	[21]
	PVX	*Sl*	DCL4 contributes to tolerance to virus infection	[30]
	PVY			
RTL1	TCV	*At*	RTL1 prevents siRNA production by cleaving dsRNA prior to DCL2-, DCL3-, and DCL4-processing	[37]
	TVCV			
	CMV			
	TYMV			

### 2.2. AGOs: Effectors of Small RNA–Guided Silencing

AGO proteins are also conserved core effectors in RNAi pathways, although their number is not the same in species from different kingdoms (animals, insects, plants). To date, ten AGO proteins (AGO1-10) have been found in *A. thaliana*, and many exert their functions in the antiviral RNAi or RNA-directed DNA methylation (RdDM) pathway [10,40]. Inoculation of single or double *ago* mutant *A. thaliana* lines with wild-type or RSS-defective viruses show that AGO1 is less effective in regulating host resistance to some plant viruses than AGO2 [8,10,13,41]. In 2017, Alazem and others reported that AGO2 and AGO3, rather than AGO1, are responsible for enhancing host resistance to bamboo mosaic virus (BMV) infection [42]. Currently, AGO4 is known to participate mainly in the RdDM pathway, in which it is guided by 24-nt siRNAs to scaffold target transcripts, thereby directing the methylation of the corresponding template DNA by recruited methyltransferases. This leads to transcriptional gene silencing of the target locus, e.g., transposable elements, which may act on geminiviral DNA. In that sense it functions as an important epigenetic defense against geminivirus infections [10,43]. Considering that the functions of AGOs in antiviral defense have been reviewed in many excellent reviews [4,10,43], we refrain from describing them in detail here.

### 2.3. Core Antiviral RNAi Effectors: RDRs and Their Helper Proteins

**Figure 3:**
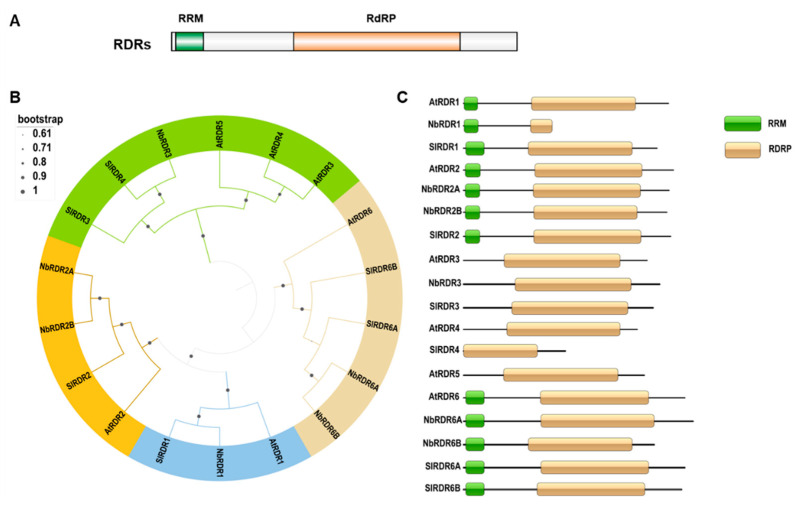
Phylogenetic relationship and conserved domains of *A. thaliana*, *N. benthamiana*, and *S. lycopersium* RDRs. (**A**) Diagram of the domain organization of the plant RDRs. (**B**) Neighbor-joining phylogenetic trees constructed after multiple alignments using *A. thaliana*, *N. benthamiana*, and *S. lycopersium* RDR protein sequences. The bootstrap values calculated using 1000 replicates are indicated. The four RDR gene families are marked with four different colors. (**C**) Schematic diagrams showing conserved domains in various RDRs. The SMART online tool (https://smart.embl.de/, accessed on 15 December 2025) was used to predict the conserved domains in the RDRs. RRM, RNA recognition motif; RdRP, RNA-dependent RNA polymerase domain.

**Figure 4:**
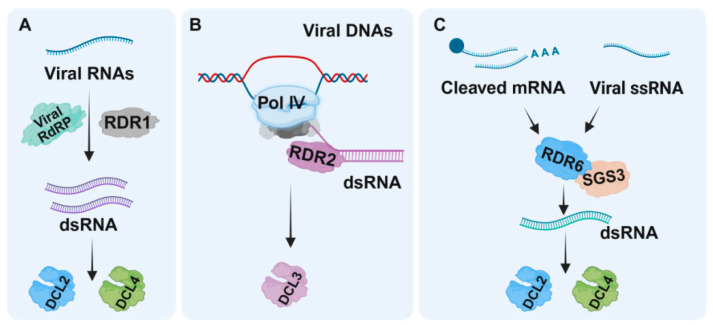
Roles of RDRs in antiviral RNA interference in plants. (**A**) Viral-encoded RdRPs generate dsRNA from viral RNA, serving as main substrates for vsiRNA production. RDR1 has been reported to amplify this process, enhancing vsiRNA production and antiviral defense [50,51,52]. (**B**) RDR2, in cooperation with Pol IV, amplifies 24-nt vsiRNAs, priming RNA-directed DNA methylation (RdDM). (**C**) RDR6, with SGS3, converts viral ssRNA and cleaved mRNA into dsRNA, boosting vsiRNA production and reinforcing RNAi-based immunity. Image created using BioRender.com, with permission. RdRP, RNA-dependent RNA polymerase; RDRs, RNA-dependent RNA polymerases; DCLs, DICER-like endonucleases; Pol Ⅳ, RNA polymerase IV; SGS3, Gene Silencing 3. Image created using BioRender.com, with permission (https://app.biorender.com/illustrations/69410c78e7e0ccf4fc0dee09, accessed on 28 December 2025).

In addition to DCLs and AGOs that perform primary effector roles, RDRs present core enzymes that contribute to an amplification of dsRNA and ultimately a larger pool of secondary siRNAs and activated RNA-induced silencing complexes (RISCs). Like viral RNA polymerases, endogenous plant RDRs can also use (aberrant) viral RNAs as templates and convert them into dsRNAs, amplifying vsiRNA production and their spread to distant plant tissues, and helping to mount a strong antiviral RNAi response [44]. Plant RDRs are known to contain an RNA recognition motif (RRM) superfamily and an RNA-dependent RNA polymerase (RdRP) domain (Figure 3A). Among the six known *A. thaliana* RDRs, RDR1, RDR2, and RDR6 are classified in the RDRα clade due to the presence of a eukaryotic RDR C-terminal catalytic DLDGD motif. RDR3, RDR4, and RDR5 are in the RDRγ clade, and these three RDRs all have an atypical catalytic DFDGD motif (Figure 3B). According to the features of the RRMs and RdRP, six *N. benthamiana* RDRs (i.e., NbRDR1, NbRDR2A, NbRDR2B, NbRDR3, NbRDR4, NbRDR6A, and NbRDR6B) have been predicted [20,45,46,47,48]. Like *A. thaliana* and *N. benthamiana*, tomato contains six predicted RDRs (i.e., SlRDR1, SlRDR2, SlRDR3, SlRDR4, SlRDR6A, and SlRDR6B) [16] (Figure 3C). To date, the functions of RDR1, RDR2, and RDR6 have been studied extensively in several plants, but the functions of RDRs in the RDRγ clade remain unclear. For example, RDR1 and RDR6 have been shown to strengthen host RNAi-based defenses against multiple DNA and RNA virus infections [9,14,15,47,48,49] (Table 2), probably through catalyzing the synthesis of additional dsRNAs from primary RISC-cleavage products. These additional dsRNAs are then converted into secondary vsiRNAs by DCL4 and DCL2 (Figure 4). RDR1 is responsible for the biogenesis of virus-induced *A. thaliana* endogenous siRNAs that can cause widespread silencing of host genes, a conserved host response to virus infections [50]. For RNA viruses, viral-encoded RdRPs, such as NIb of potyviruses, generate dsRNA replication intermediates that serve as primary substrates for vsiRNA production [51]. In a recent study, RDR1 activated by salicylic acid accelerates the production of double-stranded RNA from viral RNA, granting plants a stronger RNA silencing signal to fight invading viruses and excluding viruses from stem cells [52] (Figure 4A). RDR2 might play a significant role in amplifying 24-nt vsiRNAs generated by DCL3 to maintain and reinforce RdDM to suppress the transcriptions of DNA loci, and might also be assumed to be involved in TGS of geminiviruses [29,53] (Figure 4B). Plants silenced on, e.g., RDR1 and RDR6, show hypersusceptibility towards RNA viruses, and indicate that a strong antiviral RNAi-mediated immunity requires the RDR amplification of vsiRNAs (Figure 4C). Notably, the basal level of vsiRNAs can be detected in the *rdr1/2/6* triple mutant *A. thaliana* plants infected with an HC-Pro-deficient TuMV or a 2b-deficient CMV [9,14]. These basal vsiRNA levels may be the RDR-independent primary vsiRNAs processed from viral dsRNA replicative intermediates. However, we are unable to exclude the possibilities that (i) other host RDRs, e.g., from the γ-class, are also involved in antiviral defense; (ii) the RDR-generated endogenous siRNAs can also activate other host defense responses; and (iii) other additional unidentified vsiRNAs biogenesis pathways exist. The above hypotheses are supported by a study in which oilseed rape mosaic virus (ORMV)-infected wild-type Col-0 and *rdr126* mutant *A. thaliana* plants still exhibit similar viral siRNA accumulation profiles, although the *rdr126* mutant plants accumulate more viral genomic RNAs in the late stages of viral infection [27].

*Ty-1* and *Ty-3* are known for conferring resistance to TYLCV, which are allelic and encode for an RDR belonging to the RDRγ class, to which the *Arabidopsis* RDR3, RDR4, and RDR5 homologs belong [54]. The *Ty-1* plants infected by TYLCV exhibit neither disease symptoms nor a hypersensitive response and only accumulate low amounts of the virus [54,55]. On the other hand, *Ty*-*1*-mediated resistance can be compromised by co-replication of a betasatellite [56,57] or by cucumber mosaic virus infection [55]. After infection by TYLCV or the bipartite geminivirus–tomato severe rugose virus, hypermethylation of the viral V1 promoter region, together with higher levels of 24-nt vsiRNAs, were observed in *Ty-1* tomato plants, but not in susceptible tomato plants, indicating a role of 

*Ty-1* in TGS [55,58]. However, the mode of action of *Ty-1* in TGS has not yet been elucidated.

In the RDR-mediated RNAi pathways, SGS3, a plant suppressor of gene silencing, interacts with RDR6 to function cooperatively in the amplification of RNAi [44,48,59,60] (Figure 4C). Although SGS3 does not have RDR activity, it can function in concert with RDR6 to regulate plant growth, development, and defense. Plant *rdr6* or *sgs3* mutants display similar growth and development defects and antiviral defense impairments [44,59,61,62]. Previous studies have shown that *A. thaliana* SGS3 binds to and stabilizes RNA templates to initiate the RDR6-mediated dsRNA synthesis [63,64] to produce exogenous and endogenous siRNAs [44,53,59,60]. Although SGS3 functions in tas-siRNA- or sense-RNA-induced PTGS [44] or geminivirus-induced gene silencing [65,66,67,68], its function in some viruses is selective [23,44]. For example, *sgs3* mutants of *A. thaliana* and oilseed rape plants are susceptible to CMV infection but not to TVCV infection [69]. In addition, the accumulation level of ORMV RNA in oilseed rape plants is *SGS3* expression-dependent [69].

**Table 2:** Functions of plant RDRs and SGS3 in virus infections. CMV, cucumber mosaic virus; PSTVd, potato spindle tuber viroid; PVA, potato virus A; RDV, rice dwarf virus; RSV, rice stripe virus; TbCSV, tobacco curly shoot virus; TLCYnV, tomato leaf curl Yunnan virus; TMV, tobacco mosaic virus; TRV, tobacco rattle virus; TuMV, turnip mosaic virus; TYLCCNB, tomato yellow leaf curl China betasatellite; TYLCCNV, tomato yellow leaf curl China virus; TYLCV, tomato yellow leaf curl virus; TZSV, tomato zonate spot virus. *At*, *Arabidopsis thaliana*; *Nb*, *Nicotiana benthamiana*; *Os*, *Oryza sativa*; *Sl*, *Solanum lycopersium*.

RDR/ SGS3	Virus	Host	Main Functions	Reference
RDR1 (RDRα)	TRV	*At*	RDR1 restricts viral infection by generating secondary vsiRNAs in a cooperative manner with other RDRs	[12]
	TMV			[70]
	CMV			[9]
	TuMV	*At*	RDR1 inhibits viral infection by an unknown mechanism in a cooperative manner with other RDRs	[14]
	PSTVd	*Nb* *Sl*	RDR1 is involved in SA-mediated defense and restricts viral early systemic invasion	[71]
RDR2 (RDRα)	TRV	*At*	RDR1 restricts viral infection by generating vsiRNAs in a cooperative manner with other RDRs	[12]
	TuMV			[14]
RDR6 (RDRα)	TRV	*At*	RDR6 restricts viral infection by generating secondary vsiRNAs in a cooperative manner with other RDRs	[12]
	TMV			[70]
	TuMV			[14]
	CMV			[8,9]
	TuMV	*At*	RDR6 restricts viral systemic infection via an unclear mechanism	[14]
	RSV	*Os*		[49]
	TYLCCNV/ TYLCCNB	*Nb* *At*		[47]
	RDV	*Os*		[48]
	TbCSV	*Nb*		[67]
	TLCYnV			
	PSTVd			[72]
RDR3-5 (RDRγ)	-	*-*	-	-
Ty-1/3 (RDRγ)	TYLCV	*Sl*	Ty-1/3 encodes an RDR belonging to the RDRγ family that inhibits virus infection by transcriptional gene silencing	[54,55]
SGS3	CMV	*At*	SGS3 restricts virus infection by enhancing the production of vsiRNAs	[8,44]
	TYLCCNV/	*Nb*	SGS3 limits viral systemic infection via an unclear mechanism	[66]
	TYLCCNB			
	TbCSV			
	TLCYnV			
	PVA			[73]
	TZSV			[74]

## 3. Positive and Negative Modulators of Antiviral RNAi

### 3.1. Viral RNA Silencing Suppressors: Newly Identified Mechanisms (and Host Counter-Defense Strategies)

In plants, antiviral RNAi pathways involve cascades of consecutive events that occur in a highly coordinated manner, but are also subject to modulators upon changing physiological and abiotic/biotic stress conditions. One of the most well-known and studied modulators of antiviral RNAi are viral proteins, so-called RNA silencing suppressors (RSSs), that suppress antiviral RNAi. These proteins enable viruses to avoid being targeted by RNAi and prevent them from becoming cleared from the host, allowing them to establish a successful viral infection and stimulate their dissemination. Recent studies have uncovered new mechanisms by which viral RSSs function. For instance, Liu et al. provide insight into chloroplast immunity, an often-overlooked but crucial component of the plant immune system that works alongside RNAi to combat viral infections [75]. The study emphasizes that chloroplasts not only serve as the site of viral replication but also play a role in the recognition and suppression of viral RNAs. This underscores the interplay between cellular immune responses and antiviral RNAi pathways. For as many (consecutive) steps as there are in (antiviral) RNAi, there are as many ways that viral RSSs interfere at any of these steps and (indirectly) target core antiviral effectors, e.g., by sequestering long dsRNA and/or siRNAs, viral RSS prevent their processing by DCL, respectively, their uploading/activation into AGO-RISCs and systemic movement to more distant plant tissues. The recent findings by Tang et al. further support this notion, showing how viruses exploit the host’s proteasomal degradation machinery to inhibit RNAi and facilitate their own replication [6]. Chen et al. demonstrated that viral RNA degradation via nonsense-mediated decay (NMD) is compromised by virus–plant interactions, which can affect the efficiency of RNAi responses in certain contexts [76]. These findings highlight the complexity of plant defense mechanisms beyond the classic RNAi pathways, as viruses evolve to exploit these host mechanisms.

Collectively, viral RSSs interfere with antiviral RNAi at multiple levels by targeting distinct steps of small RNA biogenesis, amplification, effector assembly, and systemic spread (Table 3). Viral RSSs may also inhibit or target RNAi pathway machinery components for proteolytic degradation, act as targets or repressors of autophagy, inhibit the amplification of siRNAs by binding to RDRs or SGS3, or mislead the subcellular localizations of AGOs, and so on [77,78,79,80,81]. A number of excellent articles and reviews have described and discussed the functions of RSSs over the past decades, and readers are referred to these [77,78,82,83,84]. However, the field continues to progress, not only with the identification of viral proteins exerting RSS activity but also revealing as yet undiscovered strategies by which viruses, either directly by their RSS or in other ways, counteract antiviral RNAi to stimulate their multiplication, spread and (horizontal/vertical) transmission. For example, recent work has shown a novel and keen (indirect) mechanism of counter-defending antiviral RNAi by beet severe curly top virus (BSCTV). During an infection in *A*. *thaliana*, BSCTV was observed to induce the expression of an imprinted host gene *VIM5* that directly targets plant DNA methyltransferases (MET1 and CMT3) for proteasomal degradation to activate the transcription of *C2* and *C3*, which are known as repressors of RdDM [85,86]. The use/hijacking of the host protein degradation machinery (proteasomal or by autophagy) indicates that plant viruses can utilize host protein degradation machinery to inhibit the RdDM-mediated defense response to virus infections. The geminivirus-encoded C4 protein has been shown to target plant receptor-like kinases BAM1 and BAM2 (derived from barely any meristem 1 and 2) to regulate the cell-to-cell spread of RNAi [87,88]. During recent studies, new, small, functional viral proteins were also discovered with geminiviruses and potyviruses [89,90]. Among them, the TYLCV-encoded novel proteins V3 and C5, which appear not only to be involved in intra-/intercellular movement of viral entity along microfilaments to plasmodesmata but also to exert RSS activity towards suppression of TGS and PTGS [91,92,93].

Many viral RSS proteins are able to bind vsiRNAs and thereby prevent their uploading into and spread via the vascular system to distant tissues to activate systemic RNAi. Peanut clump virus (PCV) p15 is an RSS protein that acts by sequestering siRNAs, but interestingly, it has been found to dampen the systemic antiviral RNAi response by neutralizing the siRNAs by their import into peroxisomes. Upon deletion of the C-terminal part of p15, containing a peroxisomal targeting signal and needed for localization at peroxisomes, local silencing suppression of a GFP transgene was still similar as to the wild-type p15 protein. However, in the context of a viral infection, the p15 C-terminal deletion mutant only supported the accumulation of very low levels of PCV RNA in systemically infected leaves of *N. benthamiana* [94].

Another recent study initially identified the sugarcane streak mosaic virus (SCSMV) P1 protein as a classical RSS protein, able to suppress local and systemic antiviral RNAi [95]. Its mode of action is likely through sequestering dsRNA molecules (size-independently), as it was shown in another study that P1 from triticum mosaic virus (TriMV), a virus belonging to the same *Poacevirus* genus within the *Potyviridae* [96], binds to large and small dsRNA. However, and interestingly, the SCSMV P1 RSS protein can also inhibit the PVX-induced unfolded protein response (UPR) by downregulating UPR-related genes, inducing the distortion and collapse of ER polygonal meshes, and triggering cell death. The presence of a bipartite nuclear localization signal in P1, required for the nucleocytoplasmic localization distribution, appeared to be essential for RSS activity, self-interaction, UPR inhibition and cell-death induction. Further analysis revealed that the SCSMV P1 protein binds to the splicing region of ZIP60U, one of the UPR marker genes, and inhibits UPR-signaling pathways, finally leading to cell death. This is consistent with a similar cell-death response observed upon silencing of ZIP60U [95]. Although the NLS sequence plays an important role in P1 functionality, whether RSS and UPR inhibition activities are fully intertwined remains to be further investigated.

Although it is well-known that all viral suppressors of RNAi are proteinaceous, CaMV 8S RNA [39] was actually the first one to show that viral RNA molecules may be a suppressor by acting as decoys for enzymes of the RNAi pathway. Production of CaMV 8S RNA results from the premature transcription termination of incoming viral dsDNA at left-over nicks on tRNA primer binding sites, not yet fully repaired by the host DNA polymerase. Although CaMV 8S RNA represents a paradigm for RNA-based suppression of RNAi, whether additional structured viral RNAs can function as bona fide RNA silencing suppressors in plants remains incompletely explored. However, a growing number of studies report on (cytoplasmic) XRN (XRN1 in animals and XRN4 in plants)-nuclease resistant viral non-coding (nc)RNA molecules, which could not only act as sponges for components of the translational machinery but also interfere in the RNAi pathway [101,102,103]. The best example for this is flavivirus sfRNA, an ncRNA molecule resulting from XRN1-stalling at the highly structured 3′UTR sequence, which is able to suppress si- and miRNA-induced RNAi pathways in insect and animal cells by interference at the step of dsRNA cleavage by Dicer [104]. The production of XRN-resistant RNAs has been well-described and studied in only a few plant viruses. In beet necrotic yellow vein virus (BNYVV) infection, an ncRNA3 is produced to support viral long-distance movement/systemic infection of plants [97], but in an earlier study by the same group [98], it was observed that it is associated with increased levels of viral genomic RNA and protein accumulation. The additional synergistic effect of ncRNA3 on the viral p14 RSS protein suggests that this viral ncRNA molecule acts as an additional RSS molecule [98]. In the case of red clover necrotic mosaic tombusvirus, a non-coding SR1f RNA molecule of about 0.4 kb is produced from RNA1. The molecule does not result from subgenomic RNA transcription but presents a stable degradation product, resistant to XRN-digestion, and efficiently suppresses cap-dependent and -independent translation [99]. Among other so-called tombusvirus-like associated RNAs (tlaRNAs) that have been described, the most well-studied one is ST9, which encodes its own viral replicase, but relies on co-infection, typically with polerovirus, to provide the coat protein for encapsidation and transmission by aphids. The presence of ST9 has often been seen to increase symptom severity, indicating that the molecule is a virulence factor, a feature often correlated to viral suppressors of RNAi. A recent study revealed the production of a noncoding sgRNA from ST9 that results from an incomplete degradation of the genomic RNA by XRN [105]. Recently, studies on a tobacco necrosis tombusvirus 212 nt noncoding small viral (sv)RNA [100] demonstrated that this molecule is primarily produced from the 3′ terminal end of the genomic RNA1 and results from XRN-resistance. Deletion of this sequence attenuates viral infection. So far, the ability of all aforementioned XRN-resistant RNAs to interfere in antiviral RNAi has not been fully investigated in detail. XRN-resistance is known to stall ribosomal translation, but it is tempting to speculate that ncRNA molecules containing elaborate RNA structures to resist XRN1/4 degradation may also aid in virus propagation by acting as a sponge for RNAi factors, thereby suppressing (antiviral) RNAi and leading to increased virulence/symptom severity.

In the dynamic battle between viruses and their plant hosts, studies meanwhile have also identified host proteins to counter against viral RSS proteins. In a recent study, a potato type I protease inhibitor was identified to bind to the PVX p25 RSS protein and promote its degradation by autophagy and the proteasome system [106]. Similarly, Xu et al. (2025) reported a NADH dehydrogenase (ubiquinone) 1 β subcomplex subunit 9 (FaNDUFB9) from strawberry that interacts with the strawberry mottle virus (SMoV) Pro2Glu RSS protein to inhibit its expression and RSS activity [107]. In another recent study on apple stem grooving virus (ASGV), the helicase (HEL) of the viral replicase was shown to suppress local and systemic RNAi, and to have the siRNA binding ability. In the same study, a transcription factor related to ABSCISIC ACID INSENSITIVE3/VIVIPAROUS1 from pear (PbRAV1) was found to bind the HEL, and attenuate its RSS activity by interfering in the binding of siRNAs, thereby suppressing ASGV infection [108].

### 3.2. Endogenous RNA Silencing Suppressors

In 2000, a tobacco calmodulin-like protein (NtrgsCaM) was identified as an endogenous RNAi suppressor through yeast two-hybrid screens. This NtrgsCaM interacts with the HC-Pro protein of tobacco etch virus and regulates PTGS in tobacco [109]. In 2014, two independent studies showed that geminivirus infection in *N. benthamiana* and *A. thaliana* induced the expression of *NbrgsCaM* or *AtrgsCaM* (also known as *AtCML39*) to enhance disease symptoms and viral DNA accumulations [47,110]. NbrgsCaM has also been shown to suppress PTGS through the inhibition of *RDR6* expression and promotion of autophagic degradation of SGS3 [47,67]. In 2012, Nakahara and colleagues reported that NtrgsCaM could interact with different RNA virus-encoded RSSs by binding to their dsRNA-binding domains and prevent them from inhibiting RNAi [111]. Depending on the virus-pathosystem, rgsCaM thus appears to suppress PTGS and promote viral infection, or to counteract viral RSS proteins, thereby acting antiviral. It is possible that differences in interactors may determine their functional role in antiviral RNAi. Considering that the rgsCaM response to virus infection is found in different virus–plant systems [112], this indicates that the induction of rgsCaM plays an important role in the plant–virus arms race. This is supported by a study from Ghorbani et al., in which the rgsCaM promoter was shown to be inducible upon virus infection [113]. The above reports indicate that the activation of CML or rgsCaM expression by a virus challenge is an early event and may determine the outcome of infection during the interaction between plants and viruses. Another important endogenous RNAi suppressor is the ethylene-responsive element binding factor, which can down-regulate the expressions of *RDR2*, *RDR6*, *DCL2*, and *AGO2* [114].

## 4. Translational Opportunities for Antiviral RNAi in Crop Protection

Fundamental insights into antiviral RNAi have opened translational avenues for crop protection and biotechnology, enabling breeding, biotechnological, and RNA-based strategies for durable and environmentally friendly virus resistance.

### 4.1. Breeding Strategies Exploiting Natural Variation in RNAi Components

Natural allelic variation in RNAi-associated genes provides a powerful resource for breeding virus-resistant crops. A prominent example is the *Ty-1* and *Ty-3* resistance loci in tomato, which encode an RDR belonging to the γ-clade [54]. These alleles confer broad-spectrum resistance to begomoviruses by enhancing antiviral RNAi and RdDM of viral genomes [55]. Importantly, *Ty-1*/*Ty-3*-mediated resistance is quantitative rather than absolute, contributing to durable resistance without imposing strong selective pressure on viral populations [54,55]. This example illustrates how naturally occurring variation in RNAi components can be effectively exploited in breeding programs to control viral diseases in crops.

### 4.2. RNAi-Based Biotechnological Tools

RNAi has become an indispensable biotechnological tool in plant research and crop improvement. Virus-induced gene silencing (VIGS), which exploits viral vectors to trigger sequence-specific RNA silencing, is widely used for rapid functional characterization of genes in diverse plant species. Beyond classical VIGS, recent advances have expanded the scope of RNAi-based technologies. Notably, viral systems have been engineered to deliver short RNAs of defined lengths (24–32 nt), enabling targeted modulation of gene expression and epigenetic regulation in crops. Such approaches provide precise tools for functional genomics and enable manipulation of endogenous pathways without stable genome modification, increasing their applicability in plant biotechnology [115].

### 4.3. RNA-Based Antiviral Applications

In parallel, RNAi knowledge has inspired the development of RNA-based antiviral strategies that bypass genetic modification altogether. One emerging approach is the topical application of dsRNA or small RNAs to induce antiviral RNAi in plants. This strategy has been shown to confer protection against viral infection and represents a promising, environmentally benign alternative to conventional chemical treatments [53,116]. Such RNA-based applications leverage the endogenous RNAi machinery of plants and hold considerable potential for sustainable crop protection, particularly in high-value crops and controlled agricultural systems. Beyond acting solely as a trigger of RNA interference, exogenously applied dsRNA can also function as a potent elicitor of plant innate immunity. Previous work has demonstrated that dsRNA treatment induces typical pattern-triggered immunity (PTI) responses in a SERK1-dependent but DCL-independent manner, indicating that dsRNA-mediated antiviral resistance may involve RNAi-independent mechanisms [117]. Consistent with this notion, recent studies have shown that foliar application of dsRNA against plant viruses activates both sequence-specific RNAi and nonspecific PTI pathways, which can act synergistically to restrict virus accumulation [118].

Despite remaining uncertainties regarding the cellular uptake of sprayed dsRNA, its processing into non-canonical small RNAs, and the extent to which these RNAs are incorporated into Argonaute-containing effector complexes, increasing evidence supports the practical feasibility of RNA-based antiviral approaches. Several studies have reported successful foliar RNAi-based strategies for the control of plant viral diseases under experimental or semi-field conditions [116,119,120]. Together, these findings highlight the translational potential of RNA-based antiviral applications, while also underscoring the need for further mechanistic insights to optimize their efficacy, specificity, and robustness for large-scale agricultural deployment.

## 5. Conclusions

Antiviral RNAi is a central and versatile plant defense whose effectiveness depends on the coordinated activities of DCLs, AGOs and RDRs and on dynamic regulation by multiple endogenous factors. Recent work has expanded our understanding beyond canonical DCL- and RDR-functions, highlighting the significance of RDR-mediated amplification, DCL specialization, RTL1 competition, and epigenetic links to TGS. Meanwhile, viruses deploy a wide arsenal of counterstrategies—classical protein RSSs, XRN-resistant noncoding RNAs, and indirect manipulation of host degradation or methylation machinery—while plants have evolved counter-countermeasures that attenuate RSS activity. Key gaps remain: the precise molecular mechanisms of RDRγ-class proteins, the contextual roles of RTL enzymes, and the functional contributions of viral ncRNAs to RNAi suppression. Bridging these gaps will require integrated approaches combining structural biology, genetics, high-resolution small RNA profiling and cell biology. Translationally, leveraging mechanistic knowledge—through breeding for RDR/DCL alleles, engineering RNAi-enhancing factors, or developing RNA-based applications—offers promising routes for durable crop protection, but success will hinge on anticipating viral adaptability and ecological context.

## Figures and Tables

**Table 3:** Representative viral RSSs and their mechanisms of action in plants. RSSs, RNA silencing suppressors; TEV, tobacco etch virus; CMV, cucumber mosaic virus; TBSV, tomato bushy stunt virus; TCV, turnip crinkle virus; BSCTV, beet severe curly top virus; TYLCV, tomato yellow leaf curl virus; TYLCCNV, tomato yellow leaf curl China virus; PCV, peanut clump virus; SCSMV, sugarcane streak mosaic virus; CaMV, cauliflower mosaic virus; BNYVV, beet necrotic yellow vein virus; RCNMV, red clover necrotic mosaic virus; TNV-D, tobacco necrosis virus-D.

RSS	Virus	Virus Family/Genus	Primary Target(s) in RNAi Pathway	Mode of Action	Reference
HC-Pro	TEV	*Potyviridae/Potyvirus*	siRNAs, AGO1	Sequestration of siRNAs; inhibition of RISC activity	[77,78]
2b	CMV	*Bromoviridae/Cucumovirus*	AGO1, AGO4; siRNAs	Direct AGO binding; inhibition of PTGS and RdDM	[10,43,82]
p19	TBSV	*Tombusviridae/Tombusvirus*	siRNAs	Size-selective binding and sequestration of 21–22 nt siRNAs	[77,82]
P38	TCV	*Tombusviridae/Carmovirus*	AGO1	Direct interaction with AGO1, blocking slicer activity	[78,83]
C2/C3 (indirect)	BSCTV	*Geminiviridae/Curtovirus*	RdDM machinery	Indirect suppression of RdDM via host factor induction (VIM5-mediated degradation of MET1/CMT3)	[85,86]
C4	TYLCV	*Geminiviridae/Begomovirus*	Cell-to-cell RNAi spread	Targeting BAM1/BAM2 to inhibit systemic RNA silencing	[87,88]
V3, C5	TYLCV	*Geminiviridae/Begomovirus*	PTGS and TGS pathways	Suppression of both PTGS and TGS; modulation of intracellular trafficking	[91,92,93]
βC1	TYLCCNV	*Geminiviridae/Begomovirus*	RDR6 and SGS3	Suppression of RDR6 and SGS3-dependent PTGS	[47,67]
p15	PCV	*Virgaviridae/Pecluvirus*	siRNAs	Sequestration of siRNAs and relocalization to peroxisomes, impairing systemic RNAi	[94]
P1	SCSMV	*Potyviridae/Poacevirus*	dsRNA; UPR-related pathways	dsRNA binding; inhibition of UPR signaling and induction of cell death	[95,96]
8S RNA	CaMV	*Caulimoviridae/Caulimovirus*	DCL processing	RNA-based decoy that saturates DCL activity	[39]
ncRNA3	BNYVV	*Benyviridae/Benyvirus*	RNAi components (putative)	XRN-resistant ncRNA; synergizes with p14 RSS to enhance viral accumulation	[97,98]
SR1f RNA	RCNMV	*Tombusviridae/Dianthovirus*	Translation/RNA stability	XRN-resistant ncRNA; suppresses cap-dependent and independent translation	[99]
svRNA (212 nt)	TNV-D	*Tombusviridae/Tombusvirus*	RNAi factors (putative)	XRN-resistant structured RNA; deletion attenuates infection	[100]

## Data Availability

No new data were created or analyzed in this study.
